# A Broadly Cross-protective Vaccine Presenting the Neighboring Epitopes within the VP1 GH Loop and VP2 EF Loop of Enterovirus 71

**DOI:** 10.1038/srep12973

**Published:** 2015-08-05

**Authors:** Longfa Xu, Delei He, Lisheng Yang, Zhiqun Li, Xiangzhong Ye, Hai Yu, Huan zhao, Shuxuan Li, Lunzhi Yuan, Hongliu Qian, Yuqiong Que, James Wai Kuo Shih, Hua Zhu, Yimin Li, Tong Cheng, Ningshao Xia

**Affiliations:** 1 State Key Laboratory of Molecular Vaccinology and Molecular Diagnostics, School of Life Sciences, Xiamen University, Xiamen, 361102, PR China; 2 National Institute of Diagnostics and Vaccine Development in infectious diseases, School of Public Health, Xiamen University, Xiamen 361102, PR China; 3 Beijing Wantai Biological Pharmacy Enterprise Co., Ltd., Beijing, 102206, PR China; 4 Medical College, Xiamen University, Xiamen, 361102, PR China; 5 Department of Microbiology and Molecular Genetics, New Jersey Medical School, Rutgers University, 225 Warren Street, Newark, NJ 070101, USA

## Abstract

Human enterovirus 71 (EV71) and coxsackievirus A16 (CA16) are the major etiological agents of hand, foot and mouth disease (HFMD) and are often associated with neurological complications. Currently, several vaccine types are being developed for EV71 and CA16. In this study, we constructed a bivalent chimeric virus-like particle (VLP) presenting the VP1 (aa208-222) and VP2 (aa141-155) epitopes of EV71 using hepatitis B virus core protein (HBc) as a carrier, designated HBc-E1/2. Immunization with the chimeric VLPs HBc-E1/2 induced higher IgG titers and neutralization titers against EV71 and CA16 *in vitro* than immunization with only one epitope incorporated into HBc. Importantly, passive immunization with the recombinant HBc-E2 particles protected neonatal mice against lethal EV71 and CA16 infections. We demonstrate that anti-VP2 (aa141-155) sera bound authentic CA16 viral particles, whereas anti-VP1 (aa208-222) sera could not. Moreover, the anti-VP2 (aa141-155) antibodies inhibited the binding of human serum to virions, which demonstrated that the VP2 epitope is immunodominant between EV71 and CA16. These results illustrated that the chimeric VLP HBc-E1/2 is a promising candidate for a broad-spectrum HFMD vaccine, and also reveals mechanisms of protection by the neighboring linear epitopes of the VP1 GH and VP2 EF loops.

EV71 and CA16, which are small, non-enveloped viruses belonging to the genus enterovirus within the family *Picornaviridae*, are known to be the major pathogenic agents of HFMD in infants and young children^1^,^2^,^3^. HFMD is a mild self-limiting rash illness, in most cases, but EV71 and CA16 infections sometimes cause severe central nervous system complications, such as aseptic meningitis, encephalitis, poliomyelitis-like paralysis, neurogenic pulmonary edema, neurogenic cardiac failure and even death^3^,^4^,^5^,^6^. Currently, epidemics of HFMD associated EV71 and CA16 are gaining momentum^7^,^8^,^9^. Given control strategies and treatment costs, developing preventive vaccines is regarded as the most potent defense against enteroviruses, in particular for EV71 and CA16. Although, inactivated EV71 vaccines have proven effective in clinical trials^10^,^11^,^12^,^13^,^14^, no effective bivalent vaccines against EV71 and CA16 are currently being commercialized.

EV71 reportedly consists of four capsid proteins VP1, VP2, VP3 and VP4, which make it a typical member of the virus family *Picornaviridae*. Among these capsid proteins, VP1 is believed to be the major contributor to viral pathogenesis, containing important neutralizing epitopes^15^. Foo *et al*. reported that the SP70 containing amino acids 208–222 of VP1 were capable of eliciting neutralizing antibodies against EV71^16^. Residues 240–250 and 250–260 of VP1 were found to specifically react with the neutralizing monoclonal antibody 4E8^17^. In addition, researchers have successfully identified six CA16 neutralizing linear epitopes within the VP1 protein^18^. Furthermore, antisera against residues 136–150 of VP2 were found to have cross-neutralizing activity against different genotypes of the EV71 virus *in vitro*^19^. Zhao *et al*. reported that passive immunization with anti-ChiEV-A71 VLPs sera conferred full protection against lethal challenge of both EV-A71 and CVA16 infection^20^. In particular, we recently showed that immunization with the chimeric VLPs presenting VP2(aa141-155) epitope conferred potent protection against EV71 infection *in vitro* and *in vivo*^21^. However, no cross-reactive and neutralizing epitope against both EV71 and CA16 has been identified. Recently, Huang *et al*. reported that the monovalent CA16 immunized mouse sera also weakly neutralized EV71^22^, suggesting that the cross-neutralizing epitope might be present in the heterologous viruses.

In this study, we constructed a bivalent chimeric HBc-E1/2 VLPs presenting the VP1 (aa208-222) and VP2 (aa141-155) epitopes of EV71 to achieve enhanced immunogenicity and cross-protection against both EV71 and CA16 infections in a murine model. Moreover, we demonstrate that the mechanism of protection conferred by HBc-E1/2 and HBc-E2 is partly attributable to the ability to cross-neutralize and bind both EV71 and CA16 viral particles, which is an ability that was lacking in HBc-E1.

## Results

### Structure and self-assembly of chimeric VLPs displaying the VP1 (aa208-222) and VP2 (aa141-155) epitopes of EV71

**Figure 1:**
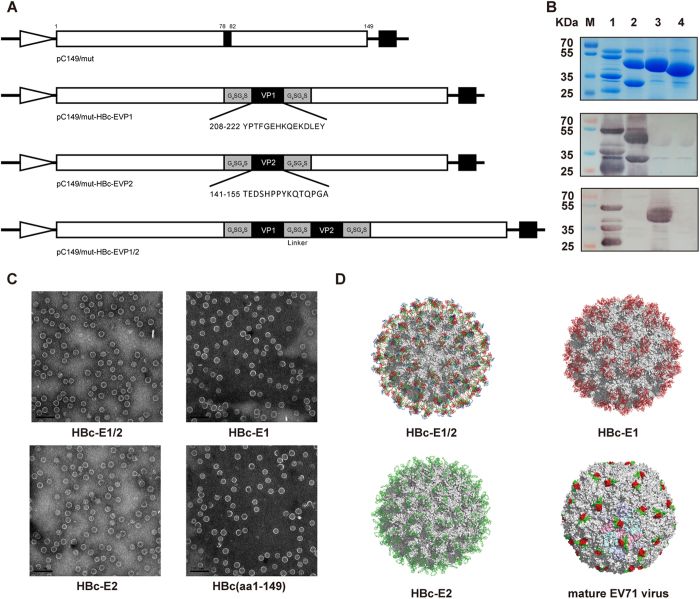
Analysis of chimeric VLPs. (**A**) Schematic presentation of the chimeric HBc protein construct. (**B**) SDS-PAGE and Western blot analyses of the *E. coli*-expressed HBc fusion proteins. Lane M, molecular mass marker; Lane 1, HBc-E1/2; lane 2, HBc-E1; lane 3, HBc-E2; lane 4, HBc (aa1-149). (**C**) Electron microscopy of HBc-E1/2, HBc-E1, HBc-E2 and HBc(aa1-149) VLPs. (**D**) Molecular modeling of chimeric VLPs and the VP1(aa208-222) and VP1(aa141-155) epitopes located on the surface of HBc VLPs colored by red and green, respectively. These two epitopes are also shown in the mature EV71 virion (PDB ID:3VBS).

Previous studies reported that immunization with the synthetic peptide SP70 of EV71-VP1 elicited cross-neutralizing antibodies *in vitro* and that vaccination with this peptide confers cross-protection *in vivo* against homologous and heterologous EV71 strains in suckling BALB/c mice^16^,^23^. We showed that immunization with the HBc-VP2 (aa141-155) particles conferred 100% *in vivo* passive protection against EV71 infection^21^. These two epitopes are located in the GH loop of VP1 and EF loop of VP2, respectively, which are exposed on the surface of the EV71 mature virus structure (PDB: 3VBS) (Fig. 1D). We sought to determine whether a bivalent chimeric VLPs vaccine presenting the VP1 (aa208-222) and VP2 (aa141-155) epitopes of EV71 would elicit a stronger immunogenic response than that elicited by VLPs containing a single epitope. In this study, the EV71-VP2 epitope (aa 141-155) and EV71-VP1 epitope (aa 208-222) were linked by two copies of a flexible decapeptide linker (G_4_SG_4_S), which was inserted into the HBc protein (amino acids 1–149) at the aa 78 and 83 sites, and expressed in *E. coli.* Accordingly, three constructs, designated HBc-E1, HBc-E2 and HBc-E1/2, were generated (Fig. 1A). To determine whether chimeric VLPs expressed the VP1 and VP2 epitopes, purified recombinant proteins were evaluated by Western blot analysis with nMAb BB1A5 and H3B10 (Fig. 1B), as well as by negative staining electron microscopy (Fig. 1C). SDS-PAGE analyses show that the molecular mass of HBc-E1/2 is slightly higher than those of HBc-E1 and HBc-E2 (Fig. 1B). Furthermore, the chimeric HBc-E1/2 VLPs were precipitated by both nMAbs BB1A5 and H3B10, suggesting the efficient presentation of VP1 and VP2 epitopes. As expected, specific reactivity with nMAb BB1A5 or H3B10 was detected for the HBc-E1 or HBc-E2 proteins, respectively (Fig. 1B). To directly confirm the efficient particle formation, the HBc-E1/2, HBc-E1, HBc-E2 and HBc (aa1-149) preparations were subjected to negative staining electron microscopy (EM). Empty particles with a diameter of 30 nm were observed for all proteins (Fig. 1C). In addition, these recombinant particles, whose structure was constructed using the crystal structure of the HBV capsid (PDB: 4G93) as a template, were located on the surface of HBc VLPs (Fig. 1D). These data demonstrate that HBc-E1/2, HBc-E1 and HBc-E2 fusion proteins self-assemble into chimeric VLPs presenting VP1 (aa208-222) and VP2 (aa141-155) epitopes.

### Immunization of BALB/c mice with HBc-E1/2 particles presenting VP1 and VP2 epitope

**Figure 2:**
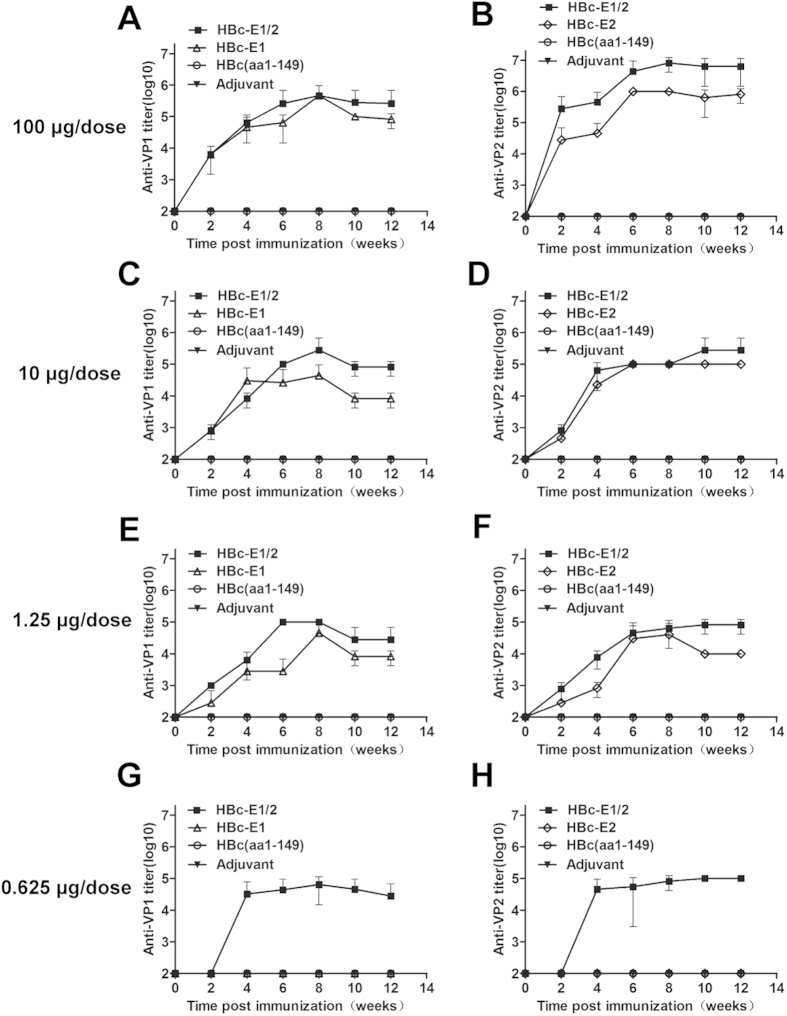
Kinetics of anti-epitope antibody responses. Groups of five mice were immunized with 0.625, 1.25, 10 and 100 μg/dose i.p. at weeks 0, 2 and 4 with HBc-E1/2, HBc-E1, HBc-E2 or HBc(aa1-149) as described in the Materials and Methods. Sera were collected at 0, 2, 4, 6, 8, 10 and 12 for the serological tests and analyzed by ELISA to measure the epitope-specific antibody response, the VP1(aa208-222)-specific antibody response (**A**,**C**,**E**,**G**) and the VP2(aa141-155)-specific antibody response (**B**,**D**,**F**,**H**). All serum samples were prepared using 10-fold dilution series, and the first dilution was 100-fold. Each point represents the mean reciprocal log10 endpoint titers and standard error.

To ascertain and compare the immunogenicity and protective properties of the VLP, both BALB/c mice and Wistar rat were used in this study. Groups of 5 female BALB/c mice were immunized with 0.625, 1.25, 10 and 100 μg/dose of one of the following samples: recombinant VLPs HBc-E1, HBc-E2, and the bivalent chimeric VLPs HBc-E1/2. Another group of mice were injected with HBc (aa1-149) as the control. The VLPs in saline were i.p. injected, with aluminum adjuvant. Booster doses were given on days 14 and 28 post-immunization. The immunized mice sera were subsequently assayed for the anti-epitope antibodies with the purified rVP1 or rVP2 protein as the antigen in the ELISA. Because we used rVP1 or rVP2 as the coated protein of the ELISA, the anti-VP1 or VP2 titer actually represented the epitope specific antibodies in the sera of recombinant VLPs immunized mice. The groups immunized with 1.25, 10 and 100 μg/dose, anti-epitope antibodies could be found in immunized mice after the primary immunization, and these antibody levels increased substantially after the booster doses were administered (Fig. 2). Figure 2A-D shows that the groups receiving 10 and 100 μg/dose chimeric VLPs HBc-E1/2 showed 2.8-fold-higher anti-VP1 antibody titers (titers ranging from 1:280,000 to 1:460,000) and 2.8-fold-higher anti-VP2 antibody titers (titers ranging from 1:280,000 to 1:8,200,000) compared to group of 0.625 and 1.25 μg/dose (Fig. 2E–H). We also tested the immunization capacity of the VLPs. The HBc-E1 and HBc-E2 antisera did not show significant immunization activity in the group receiving 0.625 μg/dose, whereas the anti-epitope titers of the HBc-E1/2 antisera increased after the second dose (anti-VP1 titers of 1:64,000 and anti-VP2 titers of 1:100,000) (Fig. 2G,H). These results demonstrated that the bivalent HBc-E1/2 VLPs were more immunogenic than HBc-E1 and HBc-E2 with a single epitope insertion, while the high dose of recombinant VLPs induced a higher antibody response than the low dose.

Group of Wistar rat immunized with 100 μg/dose of VLPs was set for comparison. In VLPs-immunized Wistar rat, the chimeric VLPs HBc-E1/2 and HBc-E1 immune sera contained reactivity for VP1 (OD450 = 1.339 and OD450 = 0.974) with a dilution of 1:100,000, whereas the anti-VP2 antibody induced by HBc-E1/2 and HBc-E2 VLP immunization include high levels of titers (OD450 = 1.862 and OD450 = 1.282) with a dilution of 1:1000,000. The data showed that levels of antibody responses against VP1 and VP2 were similar between BALB/c mice and Wistar rat groups (Supplementary Fig. S1).

### Chimeric VLPs elicit Cross-neutralizing antibodies for EV71 and CA16

We also tested the neutralization capacity of the antisera pooled for each group using an *in vitro* neutralization assay. Notably, only the high dose (10 and 100 μg/dose) of recombinant VLPs provided a detectable neutralizing antibody response against EV71 subgenotype strains. As shown in Table 1, no significant neutralizing activity was detected for the adjuvant and HBc (aa1-149) antisera (100 μg/dose group), whereas the cross-neutralization antibodies titers elicited by HBc-E1/2, HBc-E1 and HBc-E2 in mice 2 weeks after 2^nd^ booster injection against 6 EV71 subgenotype strains ranged from 1:32 to 1:256, 1:8 to 1:128 and 1:16 to 1:128, respectively. Compared to the 100 μg/dose group, the 10 μg/dose group showed lower neutralization titers ranging from 1:8 to 1:32.

The enhanced immune effect of bivalent HBc-E1/2 VLPs immunization prompted us to explore the extent of cross-neutralization and its significance on CA16 neutralization. Interestingly, the HBc-E1/2 and HBc-E2 VLPs immunized sera (100 μg/dose) could neutralize the CA16 strains of the B1b genotype, with titers ranging from 1:8 to 1:16. Moreover, the HBc-E1/2 VLPs immunized sera (10 μg/dose) showed lower neutralization titers with 1:8. Conversely, the HBc-E1 immune serum neutralized EV71 but not CA16 (Table 2). In VLPs-immunized Wistar rat, the similar neutralizing antibody titers against EV71 and CA16 were also elicited (Supplementary Fig. S1). Remarkably, our studies revealed that the EV71-VP2 epitope containing VLPs immunization provided cross-neutralization against EV71 and CA16.

### Passive immunization with the recombinant particles HBc-E1/2 protected neonatal mice against EV71 and CA16 lethal challenge

**Figure 3:**
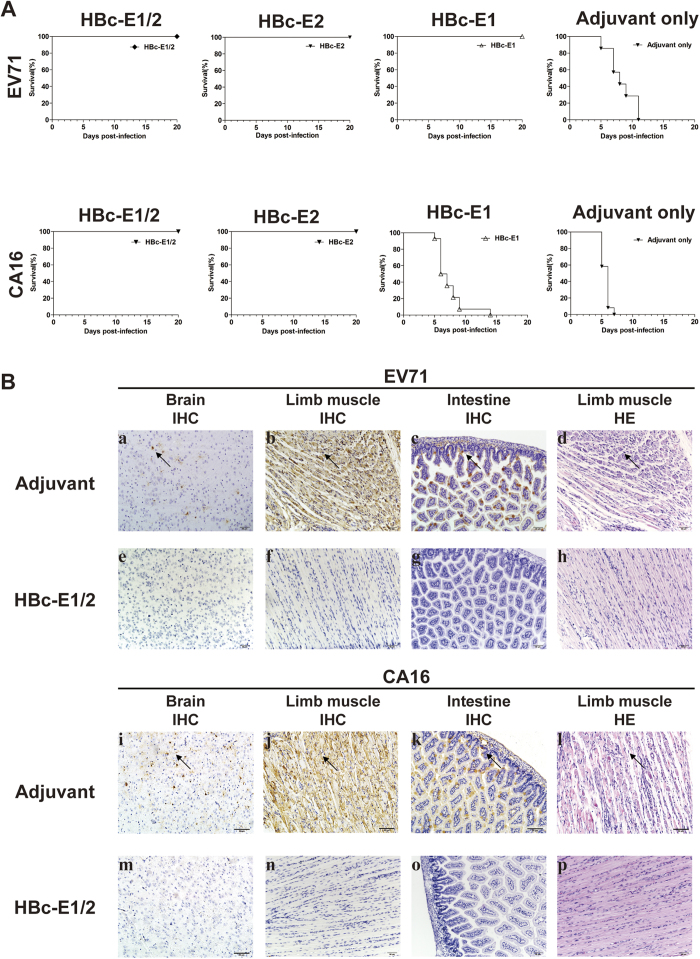
*In vivo* cross-protection of HBc-E1/2 to EV71 and CA16. (**A**) Groups of one-day old BALB/c mice born to female mice immunized with HBc-E1/2, HBc-E1, HBc-E2 or adjuvant with an immunization dose of 100 μg were challenged with pSVA-MP4 or CA16-190. The mortality were monitored and recorded daily for 20 days. The representative results of two independent experiments are shown. (**B**) Contrast of pathological changes between HBc-E1/2 group and adjuvant group. Mice born to HBc-E1/2 or adjuvant immunized female mice and challenged with pSVA-MP4 or CA16-190 were sacrificed, and the tissues were collected as described in the Materials and Methods. Representative sections analyzed by immunohistochemistry (IHC) or hematoxylin and eosin (HE) staining are shown.

Although immunization with the HBc-E1/2 could induce neutralizing antibodies against EV71 and CA16 in adult mice, it is not clear whether materal antibody could protect the neonatal mice from EV71 or CA16 induced illness and death. After the third injection, mice immunized with different dose of HBc-E1/2, HBc-E1, HBc-E2 or adjuvant were allowed to mate. The EV71 mouse-adapted virus pSVA-MP4 at a dose of 10^7^ TCID_50_ or the CA16 virus 190 at a dose of 10^5^ TCID_50_ was inoculated i.p. or i.c. to the 1-day-old mice born to the immunized mice, respectively. In the EV71 challenge test, the neonatal mice in the adjuvant group started to show illness at 4 dpi and all died within 11 dpi. One-day-old mice born to dams immunized with HBc-E2 all survived (***P < 0.0001) and were all healthy (Fig. 3A).

Moreover, in the CA16 challenge test, one-day old mice born to dams immunized with HBc-E1/2 or HBc-E2 showed a 100% survival rate (***P < 0.0001) and were all healthy throughout the experiment, while the adjuvant group started to show signs of illness 4 dpi and all died within 7 dpi (Fig. 3A). In contrast, one-day-old mice born to dams immunized with any dose of HBc-E1 all failed to survive a lethal CA16 challenge (Fig. 3A). These data suggest that immunization with HBc-E1/2 is effective to protect mice from lethal EV71 challenge and can also cross-protect against CA16.

IHC analysis and HE staining were performed to confirm the protection of HBc-E1/2 of mice challenged with EV71 or CA16. The brains (Fig. 3B,a), limb muscles (Fig. 3B,b) and intestines of the adjuvant-immunized group stained positive for EV71-specific IHC (Fig. 3B,c), while no positive stains (Fig. 3B,e,f,g) were found in the HBc-E2-immunized group. HE examination also confirmed the presence of EV71-induced severe necrotizing myositis in the limb muscles (Fig. 3B,d) in the adjuvant-immunized group compared to the normal morphology of the HBc-E2-immunized group (Fig. 3B,h). Similar results were also confirmed in the CA16 challenged mice: IHC staining of the spinal cord (Fig. 3B,i), limb muscle (Fig. 3B,j) and intestine (Fig. 3B,k) were positive, and HE staining identified severe necrotizing myositis (Fig. 3B,l) in the adjuvant-immunized group. In contrast, the HBc-E2-immunized showed no positive stains by IHC analysis (Fig. 3B,m,n,o) or pathological changes by HE staining (Fig. 3B,p). These results strongly showed that the immunization of adult female mice with HBc-E2 significantly protect the mice from lethal EV71 or CA16 challenge.

### Anti-VP2 (aa141-155), but not anti-VP1 (aa208-222), binds to CA16 authentic viral particles

**Figure 4:**
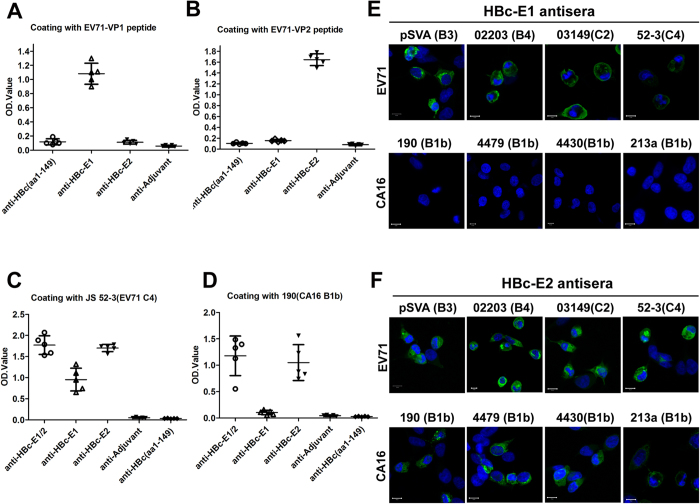
Analysis of the affinity of anti-VP2(aa141-155) and anti-VP1(aa208-222) against authentic EV71 and CA16 particles. (**A**,**B**) Anti-epitope antibody responses. (**C**,**D**) The anti-EV71 (**C**) and anti-CA16 antibody titers (**D**) of immune sera from mice immunized with VLPs. (**E**,**F**) Immunofluorescence assay of EV71 and CA16 infected RD cells. Immunized mouse sera were diluted 1:100 with PBS and then used to measure epitope-specific antibodies by ELISA with (**A**) VP1- or (**B**) VP2-derived epitope peptides as the coating antigen as described in the Materials and Methods. Each symbol represents one mouse.

To explore the mechanism of cross-neutralization against CA16 by the VP2 (aa141-155) containing VLPs antisera, we examined whether the immune antisera could bind both EV71 and CA16 authentic virions (Supplementary Table 1). The immunized mice sera were subsequently assayed for the anti-epitope antibodies with the VP1 or VP2 peptides as the antigen in the ELISA. Anti-epitope antibodies could be found in immunized mice after the last immunization with a dilution of 1:1000,000 (Fig. 4A,B). The antisera towards the VP1 and VP2-epitope were also assayed by ELISA where the cells expressing EV71 and CA16 virions were individually coated onto the 96-well microtiter plates. Both the chimeric VLPs HBc-E1/2 and HBc-E2 immune sera contained cross-reactivity for EV71 (OD450 = 1.78 and OD450 = 1.69) and CA16 (OD450 = 1.17 and OD450 = 1.04) with a dilution of 1:1000, whereas the HBc-E1 immune sera at the same dilution could only bind to EV71 (OD450 = 0.9) (Fig. 4C,D). Therefore, HBV core particles-surface displaying VP1 and VP2-epitope successfully retained the 3-dimensional conformation present in the corresponding virus particles. On the whole cell level, IFA was used to test the reactivity of HBc-E1 and HBc-E2 antisera with EV71 and CA16 strains-infected RD cells. As shown in Fig. 4E,F, all of the recombinant VLPs antisera showed good reactivity with the EV71 virus but not in non-infected cells. In contrast, HBc-E2 antisera also led to significant binding activity in CA16-infected cells, whereas HBc-E1 did not. Therefore, these data demonstrate that anti-VP2 (aa141-155), but not anti-VP1(aa208-222), could cross-react with normal EV71 and CA16 virions.

### Patient antibodies recognize the dominant cross-neutralizing epitope

**Figure 5:**
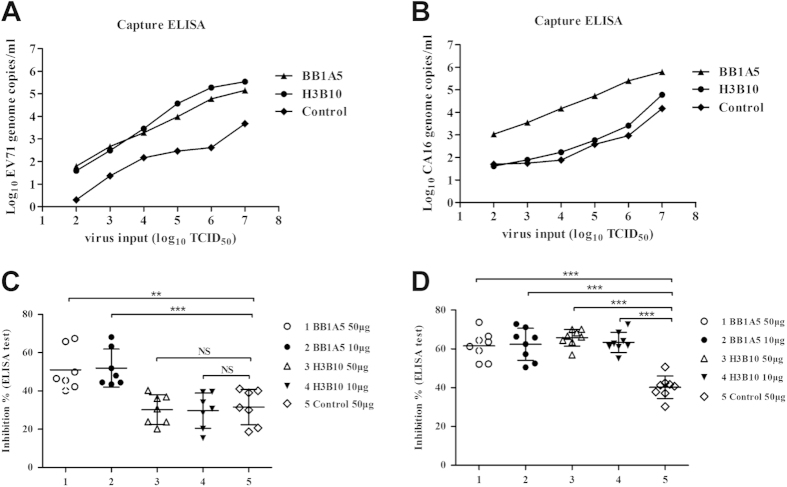
Capture ELISA and competitive ELISA test for MAbs binding to virus. (**A**,**B**) The number of RNA genome copies/ml determined by the capture ELISA and TaqMan real-time RT-PCR. The log10 values of the viral RNA copies/ml were calculated by interpolating the Ct values from the standard curve. These experiments were repeated three times and a representative result is shown. (**C**,**D**)The dilution of nMAb BB1A5 and H3B10 competed with human serum samples for specifically binding to EV71 virus 52-3 (**C**) or CA16-190 (**D**) coated on the well. A nonrelated monoclonal antibody was used as a negative or positive control, respectively.

The in-house developed VP1 (aa208-222)-specific monoclonal antibody (H3B10) and the VP2 (aa141-155)-specific nMAb (BB1A5) were tested using Capture ELISA (Fig. 5A,B). An unrelated antibody was used as a negative control for the assay. Serial dilutions of JS 52-3 with an initial concentration of 10^7^ TCID_50_ were captured by H3B10 and BB1A5. A dilution of 10^2^ TCID_50_ of the EV71 virus continued to generate a detectable signal in the real-time PCR detection assay (38.61 copies/mL and 61.79 copies/mL). Importantly, the VP2 nMAb BB1A5 also captured a viral CA16 copy number of 1073.18/mL at a dilution of 10^2^ TCID_50_.

To determine whether human antibodies specific to CA16 recognize the cross-neutralizing epitope on EV71-VP2(aa141-155), plasma was obtained from CA16-positive individuals (Supplementary Table 2). Earlier studies of eight subclinical human sera after EV71 infection that used a competitive ELISA with a VP2 epitope (nMAb BB1A5) antibody suggested that the VP2 epitope is immunodominant in human^21^. Fig. 5C shows that the nMAb BB1A5 and H3B10 equivalently inhibited the binding of patient EV71-specific antibodies to JS-52-3 (50 μg/well, 61. 6% ± 7.244 or 10 μg/well, 62.36% ± 8.264) and (50 μg/well, 65.75% ± 4.303 or 10 μg/well, 63.34% ± 5.26), respectively. Interestingly, nMAb BB1A5 also competed (50 μg/well, 51.06.26% ± 11.08 or 10 μg/well, 51.97% ± 9.936) to bind to CA16-190, whereas H3B10, an EV71-specific monoclonal antibody that recognizes the VP1 (aa208-222) epitope, did not compete to bind to CA16-190 (50 μg/well, 30.26% ± 7.773 or 10 μg/well, 29.7% ± 9.182) (Fig. 5D). These data suggest that humans, who clear EV71 or CA16 infection, develop antibodies that recognize the neutralizing epitope within the EF loop of VP2.

### Sequence alignment and structural analysis of the cross-neutralizing epitope of VP1 and VP2

**Figure 6:**
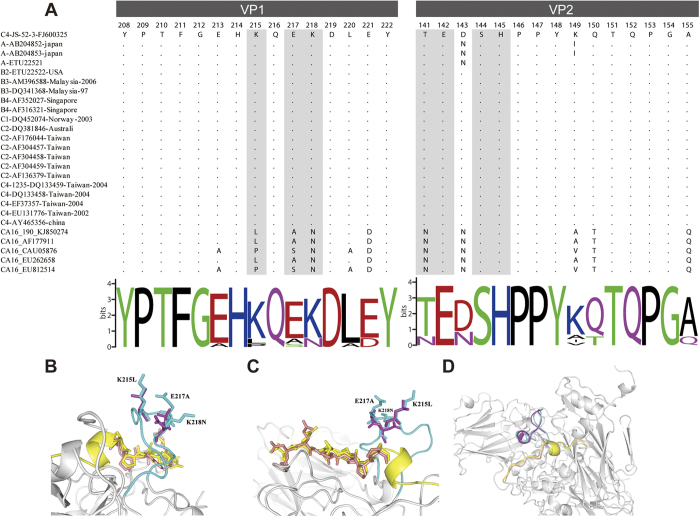
Sequence alignment of the cross-neutralizing epitope of VP1(aa208-222) and VP2(aa141-155) from NCBI database. (**A**) The quality of conservation of epitope sequence alignment and Epitopes sequence logo. The sequence logo was created using Weblogo (http://weblogo.berkeley.edu/logo.cgi). Bits represent the relative frequency of amino acids. (**B**) Comparison of the cross-neutralizing epitope of VP1 (aa208-222) and VP2 (aa141-155) between EV71 and CA16 based on molecular modeling of the mature EV71 virion structure (PDB: 3VBS). The cross-neutralizing epitope of VP2 is shown in yellows (EV71) and salmon (CA16) on the canyon region, together with the GH loop of VP1, which is in cyan (EV71) and magentas (CA16). The amino acid sequence of the EV71-VP1 GH loop differed from that of CA16 at position K215L, E217A and K218N (**A**,**B**). Stereo pictures showing the VP1 and VP2-epitope between EV71 and CA16 (**C**). All models were prepared using PyMOL (DeLano Scientific).

To investigate the mechanism underlying the cross-reactivity of the anti-HBc-E1/2 or anti-HBc-E2, but not the anti-HBc-E1, with CA16 strains, we next aligned the critical amino acid residues to further characterize the neutralization epitopes using the ClustalW2 program. The two neutralizing epitopes are shown in Weblogo format (Fig. 6A). According to the BLAST results, the epitope KQEK (highlighted in grey) was fully conserved among the strains of different EV71 sub-genogroups. In contrast, the epitope was found substantially differ in CA16 consistent with its restricted cross-reactivity. The results of our previous alanine (Ala) substitution studies suggested that the critical amino acid residues within the VP2 epitope are Thr (T141), Glu (E142), Ser (S144) and His (H145). The amino acid sequence alignment of the VP2 epitope between EV71 and CA16 showed that it is relatively well conserved with only one variant amino acid sites (residues T141N) among the critical residues. In addition, the VP2 epitope of CA16-190 (the virus used in the passive protection study) is conserved among different genotypes. This finding may also explain why the HBc-E1/2 and HBc-E2 vaccine can cross-react with normal CA16 virions.

To define the locations of the mutations and spatial relationship of these two important neutralization epitopes in the EV71 and CA16 particles, the molecular structure of the EV71^24^ was used as a template to construct the molecular structure of CA16. A view of the stereo pictures displaying the major mutations of the VP1 epitope (K215L, E217A and K218N) are shown in Fig. 6B. Rotating the image to bring the EF loop of VP2 into view (Fig. 6C) showed that the epitopes were similar and well-exposed on the surface of both EV71 and CA16. Both of these regions (Fig. 6D) have been named site 2^21^, which might contribute to a bridge that drives the separation of the VP2 αA helices.

## Discussion

Together, VP1, VP2 and VP3 constitute the outer capsid of EV71. Among them, VP1 and VP2 are the most abundant and immunodominant viral proteins and have several important neutralizing epitopes. Foo *et al.*^16^,^23^ previously showed that the peptide SP70 from amino acids 208-222 of VP1 of EV71 triggered neutralizing antibody responses when injected into mice, and the antisera from mice immunized with SP70 showed cross-antigenicity among homologous and heterologous EV71 strains. Moreover, the live recombinant attenuated Bordetella pertussis displaying SP70 on the surface stimulated a strong and sustained systemic anti-SP70 antibody response in mice^25^. In addition to strong immunogenicity, SP70 incorporated within adenovirus type 3 hexon was proven to confer protection *in vivo* to neonatal mice born to dams immunized against the lethal EV71 challenge^26^. Recently, Ye *et al.*^27^ have shown that passive immunization with anti-HBcSP55 or anti-HBcSP70 sera protected neonatal mice against lethal EV71 infections.

Conversely, the VP2-28 epitope containing residues 136–150 of VP2 was identified as another neutralizing epitope^19^, and a shorter epitope based on VP2 was then mapped to amino acids 142-146, but had non-neutralizing activity^28^. However, the ability of the neutralizing epitope of VP2 to also provide protection against EV71 infection has not yet been fully clarified. Our previous study demonstrated that HBc particles carrying the neutralizing epitope spanning amino acids 141-155 of VP2 induced immunized mice to elicit many protective antibodies, which conferred 100% *in vivo* passive protection to progeny^21^. Here, we tested whether combining these two epitopes into HBc VLPs could enhance the protective immunity. We demonstrated that the fusion of HBc with VP1 (aa208-222) and VP2 (aa141-155) linked by G_4_SG_4_S could self-assemble into VLPs with the epitopes displayed on the surface (Fig. 1). In the 0.625 μg/dose group, HBc-E1/2 induced anti-VP1 titers of 1:64,000 and anti-VP2 titers of 1:100,000 after the second immunization (Fig. 2), whereas no significant immunization activity was detected for the HBc-E1 and HBc-E2 antisera. In VLPs-immunized Wistar rat, the similar immunogenicity and protective properties were also elicited. Together, these results suggest that immunization with HBc-E1/2 with aluminum adjuvants in mice provoked markedly stronger immune responses than immunization with either the HBc-E1 vaccine or HBc-E2 vaccine.

EV71 and CA16 are the two major causative agents of HFMD. Recently, a clinical survey showed that EV71 and CA16 are responsible for 50.4% and 38.3% of the HFMD cases in China in 2009, respectively^9^. Xu *et al.* showed that approximately 21% of severe HFMD cases with neurological complications were caused by CA16 infection^29^. The development of a vaccine that protects against EV71 and CA16 has been initiated. The experimental bivalent vaccine comprising of inactivated EV71 and CA16 has been demonstrated to induce balanced protective immunity against both EV71 and CA16^22^. In our study, we found that anti-HBc-E1/2 and anti-HBc-E2 sera (10 and 100 μg/dose), but not HBc-E1, could cross-neutralize the CA16 strains of the B1b genotype and the G10 strain belonging to the A genotype, with titers ranging from 1:8 to 1:16 (Table 1). The *in vivo* protective efficacy of the chimeric VLP vaccines was assessed in EV71 and CA16 virus challenge models. Importantly, passive immunization with the recombinant particles HBc-E1/2 or HBc-E2 protected neonatal mice against lethal EV71 and CA16 challenge, while immunization with HBc-E1 failed to protect the mice (Fig. 3A). Therefore, we conclude that bivalent chimeric VLPs HBc-E1/2 is a promising candidate for a broad-spectrum HFMD vaccine.

Cross-reactivity and recombination during natural infection and circulation have been observed among enteroviruses^30^,^31^. A previous study demonstrated that EV71 and CA16 had a common antigen that was detectable by immunofluorescence^32^. Indeed, a commercial anti-EV71 MAb, MAb979, can cross- recognize and neutralize CA16 viruses^19^. In addition, Wu *et al.* showed that both humoral and cellular immunities induced by CA16 could cross-react with EV71 and that the common epitopes are likely located on the outer capsid proteins^33^. To explore the mechanism of cross-neutralization against CA16 by the VP2 (aa141-155) containing VLPs antisera, we clearly demonstrated with IFA and Capture ELISA that anti-VP2 (aa141-155), but not anti-VP1 (aa208-222), could cross-react with both normal EV71 and CA16 virions (Figs 4 and 5). This finding may partially explain the inability of anti-VP1 (aa208-222) to cross-neutralize and protect mice from lethal CA16 challenge. A sequence comparison analysis shows that the VP2 epitope is relatively well conserved with only one variant amino acid site (residues T141N) among the critical residues, which may explain the ability of the HBc-E1/2 and HBc-E2 vaccines to cross-react with normal CA16 virions.

A three-dimensional structural characterization for the neutralizing epitopes that can support an effective vaccine was developed. We used the available EV71 crystal structures as a template to construct the molecular structure of CA16. In this model, the VP2 (aa141-155) epitope appeared to lie close to the VP1 GH loop (residues 208–222), and it was exposed on the surface of EV71 and CA16 (Fig. 6). Recently, cryo-EM analysis also revealed that the two identified linear neutralizing epitopes are well preserved on the surfaces of EV71 and CA16 VLPs^34^. We speculate that the EF loop of VP2 and the GH loop of VP1 and VP3, named site 2, might contribute to a bridge that drives the separation of the VP2 αA helices, while the larger loop might also allow the exit of RNA in EV71 and CA16. In support of this speculation, the CA16 135S-like structure suggests that the RNA may exit through the enlarged two-fold axis channel^35^. Moreover, cryo-electron microscopy reconstructions of the EV71 A-particle suggest that the icosahedral two-fold axis opens a channel that acts as a gateway in the viral capsid, regulating the release of genomic material from the altered particle^36^. Further cryo-EM studies of the interactions of the virus with VP2 antibodies may provide a precise mechanism of cross-neutralization by the VP2 epitope.

Multi-epitope vaccines based on neutralizing epitopes of EV71 and CA16 should be considered in future studies to develop better epitope vaccines. In addition, testing different carriers and adjuvants will contribute to the improvement of immunity and protection of epitope vaccines. In addition to identifying neutralizing epitopes of other enteroviruses that cause HFMD, universal HFMD recombinant vaccines may be able to prevent wild enterovirus transmission in endemic countries.

## Methods

### Ethics statement

All animal experiments were carried out in accordance with the guidelines of the Xiamen University Institutional Committee for the Care and Use of Laboratory Animals and were approved by the Xiamen University Laboratory Animal Management Ethics Committee. Written informed consent was obtained from the donor for use of the serum sample. Independent Ethics Committee approval was obtained from the Ethics Committee of the National Institute of Diagnostics and Vaccine Development in infectious diseases.

### Construction of recombinant plasmids

The recombinant HBc-VP2(aa141-155) (designated HBc-E2) and pC149/mut plasmid vectors were prepared as described previously^21^. To express HBc-E1 fusion protein, VP1(aa208-222) DNA sequences were inserted into pC149/mut plasmids using annealing oligos (E1-F, GATCCTATCCCACATTCGGAGAACACAAACAGGAGAAAGATCTTGAATATG; E1-R, AATTCATATTCAAGATCTTTCTCCTGTTTGTGTTCTCCGAATGTGGGATAG) to generate the HBc-E1 plasmid, which inserted 15 amino acids between aa78 and 83 of pC149/mut. To express the chimeric VLPs HBc-E1/2, the coding sequence of the peptide linker G_4_SG_4_S was introduced into the regions between the VP1(aa208-222) and VP2(aa141-155) genes, which was inserted into the pC149/mut plasmid vector using the annealing oligos (E1/2-F, GATCCTATCCCACATTCGGAGAACACAAACAGGAGAAAGATCTTGAATATGGTGGTGGTGGTAGTGGTGGTGGTGGTAGTACGGAAGATAGTCACCCCCCTTACAAGCAGACTCAACCCGGCGCCG; E1/2-R, AATTCGGCGCCGGGTTGAGTCTGCTTGTAAGGGGGGTGACTATCTTCCGTACTACCACCACCACCACTACCACCACCACCATATTCAAGATCTTTCTCCTGTTTGTGTTCTCCGAATGTGGGATAG). The annealed oligos were designed to create BamHI and EcoRI overhangs at the two ends and can be used immediately in a ligation reaction as described previously^21^. The recombinant plasmids were verified by sequencing. The recombinant HBc-E1, and HBc-E2 HBc-E1/2 proteins were expressed and purified as outlined in the Supplementary Methods.

### Vaccine preparation and immunization of mice

To compare the recombinant protein vaccines, we equalized their protein content in all vaccine preparations. These proteins were diluted in PBS to the desired final concentrations and mixed with an equal volume of aluminum adjuvant (amorphous aluminum hydroxyphosphate sulfate).

Female BALB/c mice and Wistar rat (purchased from Shanghai SLAC Laboratory Animal Co. Ltd, Shanghai) aged 6–8 weeks were used in the immunization experiments. Groups of 5 mice were immunized with 0.625, 1.25, 10 and 100 μg/dose of one of the following samples: Adjuvant, HBc (aa1-149), HBc-E1, HBc-E2 and HBc-E1/2. Adjuvant or HBc (aa1-149) served as a negative control. Each mouse was intraperitoneally (i.p.) injected with 0.5 mL of the samples and received the same dose of booster injection after 2 weeks. The immunized animals were bled at 0, 2, 4, 6, 8, 10 and 12 weeks for the serological tests, and the serum was collected and stored at −80 °C. Then, the serum and strains used for ELISA, IFA and neutralization assays are described in the Supplementary Data.

### Sequence alignment and structure homology prediction

The NCBI PubMed website (http://www.ncbi.nlm.nih.gov/pubmed/) was searched for the genome sequences of all related EV71 and CA16 strains. The sequences of the VP1 (aa208-222) and VP2 (aa141-155) epitopes were aligned using the ClustalW2 program. A sequence logo was created using Weblogo (http://weblogo.berkeley.edu/logo.cgi). The three-dimensional structural models of the chimeric VLPs and CA16 were generated via homology modeling with the crystal structure of the HBV capsid (PDB: 4G93) and mature EV71 virion (PDB ID:3VBS) as the template.

### Lethal challenge test

Immunized female BALB/c mice aged 6–8 weeks were allowed to mate 2 weeks after 2^nd^ booster injection. The neonatal mice were born and then challenged i.p. with 10^7^ TCID_50_ of EV71 mouse-adapted strain pSVA (from an infectious clone of EV71 strain SK-EV006, GenBank NO. AB469182) or intracranially (i.c.) with 10^5^ TCID_50_ of CVA16 strain 190 within 24 h after birth. Every group contained two independent experiments (n = 13–14). Mice were monitored daily for body weight, clinical illness and death until day 20-post infection. The grade of clinical disease was scored as follows: 0, healthy; 1, lethargy and inactivity; 2, wasting; 3, limb weakness; 4, hindlimb paralysis; and 5, moribund and death. Statistical analyses were performed as outlined in the Supplementary Methods. The histopathologic and immunohistochemical analyses as described in the Supplementary Data.

## Additional Information

**How to cite this article**: Xu, L. *et al.* A Broadly Cross-protective Vaccine Presenting the Neighboring Epitopes within the VP1 GH Loop and VP2 EF Loop of Enterovirus 71. *Sci. Rep.* **5**, 12973; doi: 10.1038/srep12973 (2015).

## Supplementary Material

Supplementary Dataset 1

Supplementary Information

## Figures and Tables

**Table 1:** Neutralization capacity of the pooled antisera against EV71 viruses.

Pooled antisera	**100 μg/dose**					**10 μg/dose**				
	**pSVA**	**02203**	**03149**	**52-3**	**02969**	**pSVA**	**02203**	**03149**	**52-3**	**02969**
	**(B3)**	**(B4)**	**(C2)**	**(C4)**	**(C5)**	**(B3)**	**(B4)**	**(C2)**	**(C4)**	**(C5)**
Anti-HBc-E1/2	1:32	1:64	1:128	1:256	1:128	1:32	1:32	1:16	1:32	1:16
Anti-HBc-E1	1:8	1:16	1:64	1:64	1:128	1:8	1:16	1:32	1:16	1:8
Anti-HBc-E2	1:16	1:16	1:32	1:128	1:64	1:8	1:8	1:16	1:8	1:8
Anti-HBc(aa1-149)	<1:8	<1:8	<1:8	<1:8	<1:8	<1:8	<1:8	<1:8	<1:8	<1:8
adjuvant	<1:8	<1:8	<1:8	<1:8	<1:8	<1:8	<1:8	<1:8	<1:8	<1:8

**Table 2:** Neutralization capacity of the pooled antisera against CA16 viruses.

Pooled antisera	**100 μg/dose**					**10 μg/dose**				
	**190**	**4479**	**4430**	**213a**	**G10**	**190**	**4479**	**4430**	**213a**	**G10**
Anti-HBc-E1/2	1:16	1:16	1:16	1:16	1:8	1:8	1:8	<1:8	1:16	<1:8
Anti-HBc-E1	<1:8	<1:8	<1:8	<1:8	<1:8	<1:8	<1:8	<1:8	<1:8	<1:8
Anti-HBc-E2	1:8	1:16	1:16	1:8	1:8	<1:8	<1:8	<1:8	1:8	<1:8
Anti-HBc(aa1-149)	<1:8	<1:8	<1:8	<1:8	<1:8	<1:8	<1:8	<1:8	<1:8	<1:8
adjuvant	<1:8	<1:8	<1:8	<1:8	<1:8	<1:8	<1:8	<1:8	<1:8	<1:8

The lowest serum dilution tested was 1:8. Antibody titers were determined using a *in vitro* assay, as described in the study. Antisera were collected at 2 weeks after 2^nd^ booster injection.
