# Supplementary material for: A Broadly Cross-protective Vaccine Presenting the Neighboring Epitopes within the VP1 GH Loop and VP2 EF Loop of Enterovirus 71
Source: Sci Rep. 2015 Aug 5;5:12973. doi: 10.1038/srep12973 (PMC4525384; doi:10.1038/srep12973)
Supplement: Supplementary Dataset 1 [file srep12973-s1.doc]

**A Broadly Cross-protective Vaccine Presenting the Neighboring Epitopes within the VP1 GH Loop and VP2 EF Loop of Enterovirus 71**

Longfa Xu^b,#^, Delei He^a,#^, Lisheng Yang^a^, Zhiqun Li^a^, Xiangzhong Ye^c^, Hai Yu^b^, Huan zhao^b^, Shuxuan Li^a^, Lunzhi Yuan^a^, Hongliu Qian^d^, Yuqiong Que^a^, James Wai Kuo Shih^b^, Hua Zhu^e^, Yimin Li^c^, Tong Cheng^a,b*^, Ningshao Xia^a,b **^

**
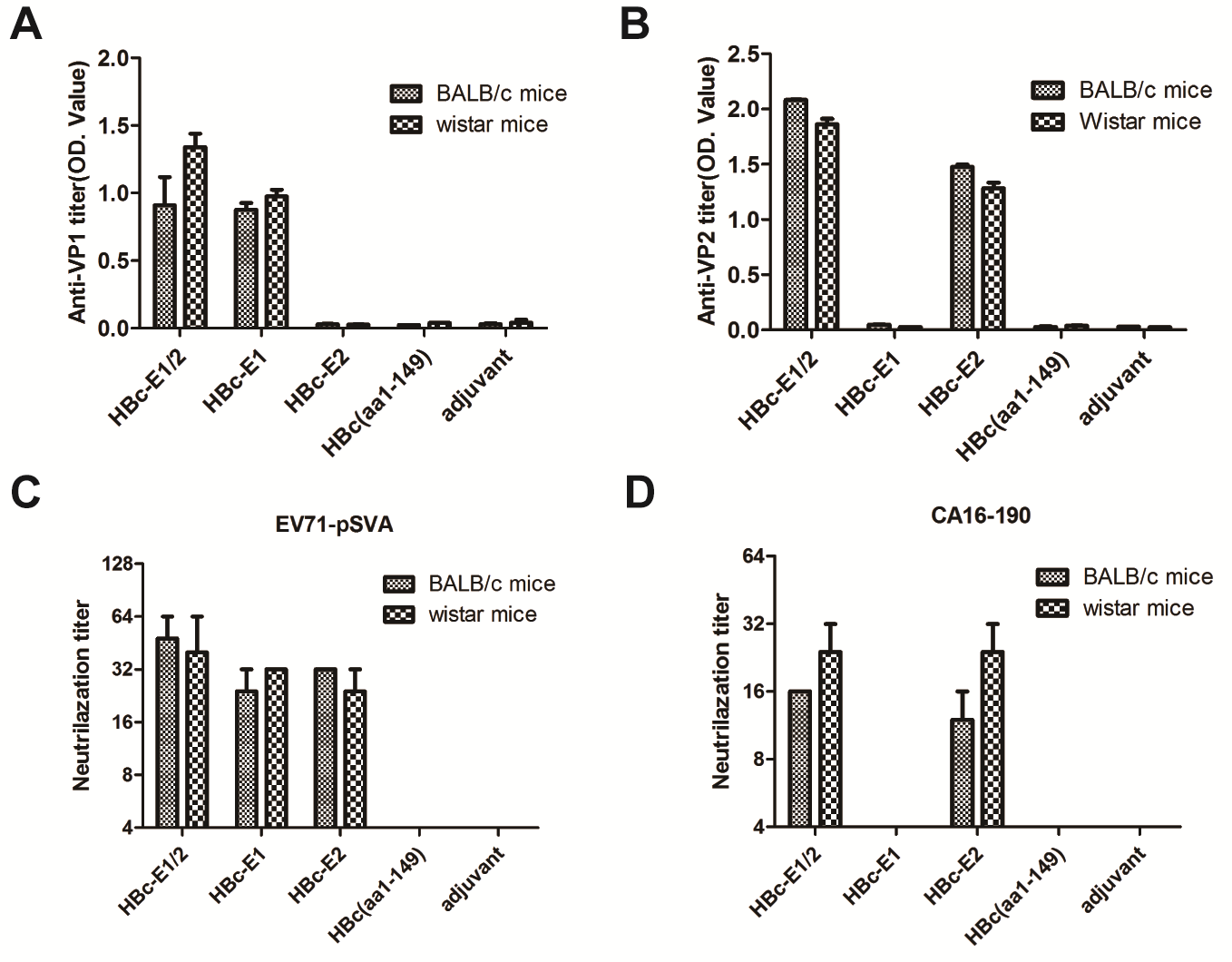
**

**Supplementary Fig. S1.** Comparison of the antigenicities and neutralizing antibodies responses in BALB/c mice and Wistar rat immunized with chimeric HBc-E1/2, HBc-E1 and HBc-E2 proteins. Wistar rat serum containing anti-VP1 (A) and VP2 (B) antibodies were added, recombinant HBc(aa1-149) was used as a negative control. Anti-EV71 neutralization titers (C) and anti-CA16 neutralization titers (B) of VLPs immune sera at two weeks after the last immunization were measured by *in vitro* neutralization assay.

**Supplementary Table 1.** The information of related enterovirus strains

| **Virus strain** | **Genotype** | **Origin** | **Year** | **Genbank No.** |
| --- | --- | --- | --- | --- |
| pSVA ^a^ | EV71 B3 | Tokyo | 1997 | AB469182 |
| 02203 | EV71 B4 | Taiwan | 2006 | JF420549 |
| 03149 | EV71 C2 | Taiwan | 2008 | JF420552 |
| 52-3 | EV71 C4 | Jiangsu | 2006 | FJ600325 |
| 02969 | EV71 C5 | Taiwan | 2008 | JF420554 |
| 190 | CA16 B1b | Taiwan | 2007 | KJ850274 |
| 4479 | CA16 B1b | Xiamen | 2010 | JX127277 |
| 4430 | CA16 B1b | Xiamen | 2009 | JX127274 |
| 213a | CA16 B1b | Xiamen | 2009 | JX127259 |
| G10 | CA16 A | USA | 1951 | U05876 |

^a^ pSVA, SK-EV006/Malaysia/1997, was sourced from the Tokyo Metropolitan Institute for Neuroscience of Japan.

**Supplementary Table 2.** The PCR detection and neutralization assay of human serum samples from patients with CA16

| **No. of sera** | **PCR detection** | | **Neutralization titer** | |
| --- | --- | --- | --- | --- |
|  | **CA16** | **EV71** | **EV71** | **CA16** |
| 1 | + | - | 0 | 2048 |
| 2 | + | - | 0 | 256 |
| 3 | + | - | 0 | 512 |
| 4 | + | - | 0 | 4096 |
| 5 | + | - | 0 | 2048 |
| 6 | + | - | 0 | 256 |
| 7 | + | - | 0 | 4096 |

**Supplementary Methods**

**Cells and viruses.**

RD cells obtained from American Type Culture Collection (ATCC) were cultivated in Minimal Essential Medium (MEM, GIBCO) supplemented with 10% FBS (PAA) plus 2mM L-glutamine, 100U of penicillin, and 100 μg of streptomycin per ml, and were used for virus preparation, titration and neutralization assays. EV71 and CA16 strains were respectively isolated from throat swabs and anal swabs of HFMD patients from the Jiangsu, Taiwan and Xiamen (Supplementary Table 1). The EV71 or CA16 virions were loaded onto a 15-50% continuous sucrose gradient to produce fractions with densities at 20-40% after ultracentrifugation for 3 h (32,000 × g, SW41Ti rotor, Beckman). The collected fractions were pelleted (100,000 × g for 2 h) and then resuspended in PBS. The protein content of the purified virus was measured by the BCA protein assay (Bio-Rad) then stored in a −80℃ freezer.

**Expression and purification of** **recombinant particles.**

For cloning and expression experiments, *E. coli* strains DH5α and ER2566 (NEB, American) were used. The recombinant HBc-E1, and HBc-E2 HBc-E1/2 proteins were expressed and purified as described previously and analyzed by negative staining electron microscopy. The concentrations of each of the recombinant HBc antigens were determined using a BCA protein assay, and antigens were saved at −20℃.

**SDS-PAGE and Western blotting.**

To analyze the purified fusion proteins, samples were subjected to 12% SDS-PAGE and Western blotting as described previously [^1^](#_ENREF_1) with the following modifications. Briefly, the transferred nitrocellulose membrane was incubated in blocking solution (5% skim milk in Tris-buffered saline, TBS) for 1 h at 37℃, and washed thrice with TTBS (0.05% Tween 20 in TBS) followed by incubation at 37℃ for 1 h with anti-VP1(aa208-222) nMAb H3B10 [^3^](#_ENREF_3) and anti-VP2(aa141-155) nMAb BB1A5 [^1^](#_ENREF_1) that were prediluted to 1:1,000 with blocking solution, and washed thrice times with TTBS. Protein bands were reacted in turn with MAb diluted 1:5,000, followed by incubation with alkaline phosphatase (AKP)-conjugated goat anti-mouse IgG (DAKO).

**ELISA.**

The specific IgG responses in serum samples were determined in accordance with the previously described methodology [^1^](#_ENREF_1). Briefly, the 96-well plates were coated with 1 μg/well of VP1 or VP2 peptides in 50 mmol/L carbonate buffer (pH9.6), followed by incubation at 4℃ for 12 h. After washed with PBS containing 0.05% Tween 20, the plates were blocked with 0.05% Tween 20 and 1% bovine serum albumin in PBS for 2 h at 37℃. The serum samples using 10-fold dilution series, and the first dilution was 100-fold, added to the wells, and were applied for 30 min at 37℃, and then incubated with Horseradish peroxidase-conjugated goat anti-mouse (GAM-HRP) IgG antibody/well in a 1:5,000 dilution for 30 min at 37℃. After color development, absorption was measured at A_450/620_.

**Competitive ELISA.**

The procedure adopted for the competitive ELISA is the same as that for recombinant antigens ELISA. First, the nMAb H3B10 and BB1A5 (10 or 50 μg/well) were added to the wells coated with JS-52-3 or CA16-190 in blocking solution (final volume: 50 μL/well), and the plates were incubated for 30 min at 37℃. Human serum (1:50 dilution) was then added and incubated at 37℃ for 30 min. After the washings, mouse anti-human antibody labeled with horseradish peroxidase was added to the plates (100 μL/well) at a 1:200 dilution in PBS with 1% BSA (bovine serum albumin), and incubated for 30 min at 37℃. The OD value was converted to percentage inhibition (PI) using the following formula: PI (%) =100−[(OD_sample_/OD_control_)×100], where the OD of the control well represents the well containing only human serum.

**Clinical human serum serum samples**

Well-characterized clinical human serum samples collected from EV71 or CA16 infected patients were obtained from the previous studies . Each serum sample was confirmed by RT-PCR and neutralizing antibody titer for EV71 or CA16 (Table 2). The EV71 serum samples were previously published [^1^](#_ENREF_1). All serum samples were kept in −20℃ freezer.

***In vitro* Neutralization assay**

Strains used for neutralization assays are described in the Supplementary Data. RD cell monolayers were diluted in MEM supplemented with 2% FBS and then seeded into 96 well plates (NUNC) ( 10,000 cells per well). Serum samples were heat-inactivated at 56℃ for 30 min followed by diluting in MEM by two-fold serial dilutions ranging from 1:8 to 1:4,096 . Each sample was challenged with 100 TCID_50_ per well of EV71 or CA16 strain. The mixed samples were incubated at 37℃, 5% CO_2_ for 1 h and then incubated with the prepared RD cells in 96 well plates. The cultures were incubated at 37℃ and observed for the CPE phenomenon in infected cells for 7 days. The neutralization titers were calculated according to the highest dilution in over 50% CPE, taken as the average of the triplicates.

**Immunofluorescence Assay (IFA)**

24-well plates were pre-covered with 13×13 mm glass coverslips, seeded with RD cells and infected with EV71 or CA16 strains. After incubating at 37℃ for 12 h, cells on the coverslips were fixed (4% paraformaldehyde, 30 min) and permeabilized (PBS+0.3% Triton X-100, 10 min). After blocking with goat serum for 1 h, the cells were incubated with HBc-VP1(aa208-222) and HBc-VP2(aa141-155) antisera (1:200 dilution) at 37℃ for 1 h. Then the cells were washed and incubated with GAM-FITC 37℃ for 30 min. At last, the cells were washed and stained with DAPI (5 min). The fluorescence of the cells on the coverslips was observed by confocal microscopy (MRC-1024, Biorad, Hercules, CA).

**Histopathologic and immunohistochemical analyses.**

Challenged mice born to the adjuvant immunized group or HBc-E2 immunized group between day 6 and day 8 post-infection were subjected to histopathologic and immunohistochemical analyses as described previously [^1^](#_ENREF_1). The primary antibodies were the I2D7 mouse anti-EV71 VP1 or 7D10 anti-CVA16 VP1 monoclonal antibodies (1 mg/mL, 1:1,000 dilution).

**Statistical analysis.**

A statistical analysis was performed using the Graphpad Prism 5 software (Graphpad Software, Inc.,USA). The survival curves were calculated using the Mantel Cox Log-rank test. The inhibition rate of the competitive ELISA was calculated by the paired t-test. The health scores are shown as means. The competitive ELISA data are shown as means ±standard deviations (SD). A P value of < 0.05 was considered as statistically significant.

**Supplementary References**

1. Xu, L. *et al.* Protection against Lethal Enterovirus 71 Challenge in Mice by a Recombinant Vaccine Candidate Containing a Broadly Cross-Neutralizing Epitope within the VP2 EF Loop. *Theranostics* **4**, 498-513, (2014).

2. Yang, H. J. *et al.* Expression and immunoactivity of chimeric particulate antigens of receptor binding site-core antigen of hepatitis B virus. *World J Gastroenterol* **11**, 492-497, (2005).

3. Chen, Y. *et al.* Antigenic analysis of divergent genotypes human Enterovirus 71 viruses by a panel of neutralizing monoclonal antibodies: current genotyping of EV71 does not reflect their antigenicity. *Vaccine* **31**, 425-430, (2013).

4. Xu, F. *et al.* Development of an IgM-capture ELISA for Coxsackievirus A16 infection. *J Virol Methods* **171**, 107-110, (2011).

5. Xu, F. *et al.* Performance of detecting IgM antibodies against enterovirus 71 for early diagnosis. *PLoS One* **5**, e11388, (2010).
