# Supplementary material for: A Broadly Cross-protective Vaccine Presenting the Neighboring Epitopes within the VP1 GH Loop and VP2 EF Loop of Enterovirus 71
Source: Sci Rep. 2015 Aug 5;5:12973. doi: 10.1038/srep12973 (PMC4525384; doi:10.1038/srep12973)
Supplement: Supplementary Information [file srep12973-s2.doc]

**A Broadly Cross-protective Vaccine Presenting the Neighboring Epitopes within the VP1 GH Loop and VP2 EF Loop of Enterovirus 71**

Longfa Xu^b,#^, Delei He^a,#^, Lisheng Yang^a^, Zhiqun Li^a^, Xiangzhong Ye^c^, Hai Yu^b^, Huan zhao^b^, Shuxuan Li^a^, Lunzhi Yuan^a^, Hongliu Qian^d^, Yuqiong Que^a^, James Wai Kuo Shih^b^, Hua Zhu^e^, Yimin Li^c^, Tong Cheng^a,b*^, Ningshao Xia^a,b **^

**
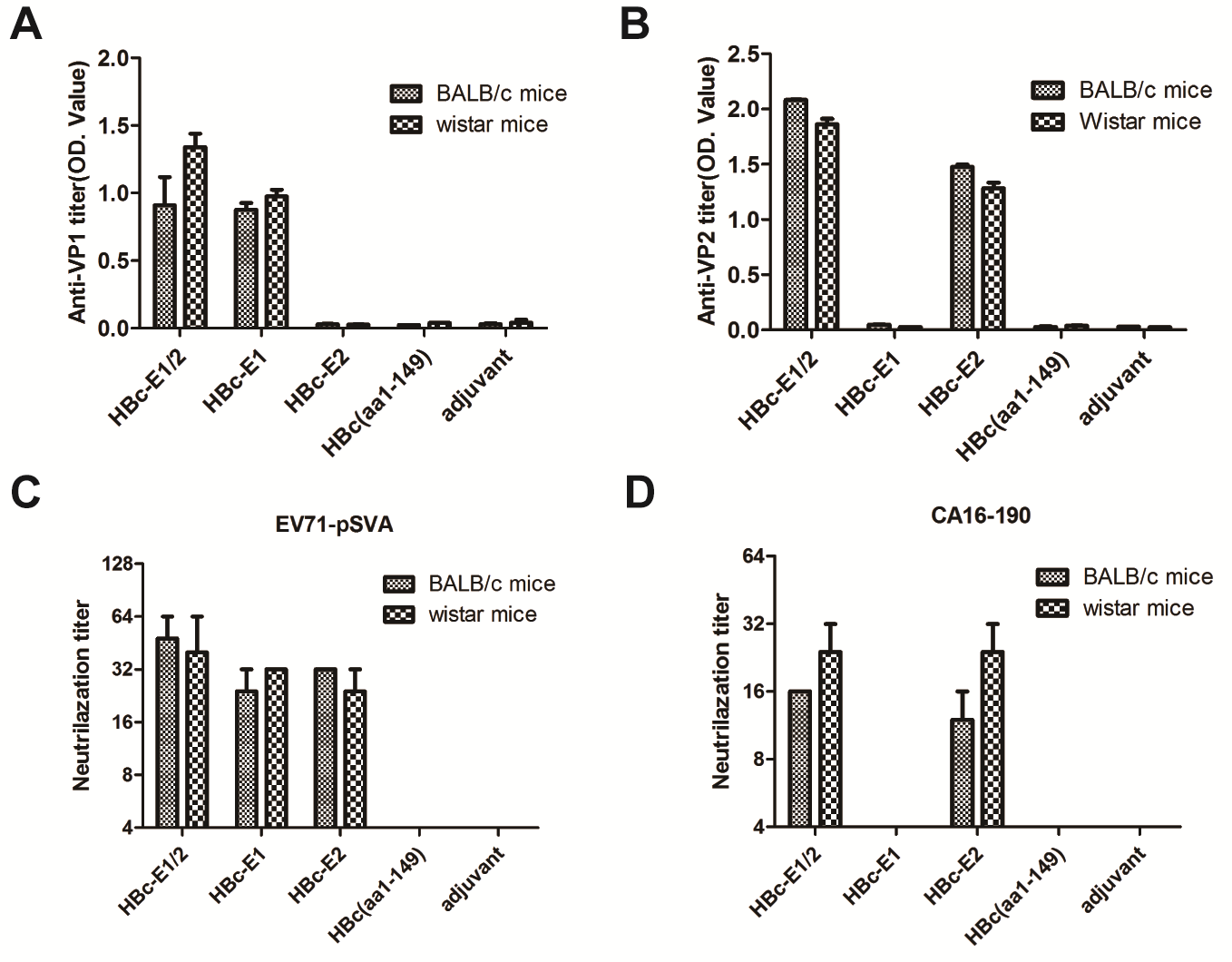
**

**Supplementary Fig. S1.** Comparison of the antigenicities and neutralizing antibodies responses in BALB/c mice and Wistar rat immunized with chimeric HBc-E1/2, HBc-E1 and HBc-E2 proteins. Wistar rat serum containing anti-VP1 (A) and VP2 (B) antibodies were added, recombinant HBc(aa1-149) was used as a negative control. Anti-EV71 neutralization titers (C) and anti-CA16 neutralization titers (B) of VLPs immune sera at two weeks after the last immunization were measured by *in vitro* neutralization assay.
